# Effects of Physical Exercise on Autophagy and Apoptosis in Aged Brain: Human and Animal Studies

**DOI:** 10.3389/fnut.2020.00094

**Published:** 2020-07-28

**Authors:** Diana Zukas Andreotti, Josiane do Nascimento Silva, Amanda Midori Matumoto, Ana Maria Orellana, Paloma Segura de Mello, Elisa Mitiko Kawamoto

**Affiliations:** ^1^Laboratory of Molecular and Functional Neurobiology, Department of Pharmacology, Institute of Biomedical Sciences, University of São Paulo, São Paulo, Brazil; ^2^Laboratory of Molecular Neuropharmacology, Department of Pharmacology, Institute of Biomedical Sciences, University of São Paulo, São Paulo, Brazil

**Keywords:** aging, exercise, autophagy, apoptosis, brain

## Abstract

The aging process is characterized by a series of molecular and cellular changes over the years that could culminate in the deterioration of physiological parameters important to keeping an organism alive and healthy. Physical exercise, defined as planned, structured and repetitive physical activity, has been an important force to alter physiology and brain development during the process of human beings' evolution. Among several aspects of aging, the aim of this review is to discuss the balance between two vital cellular processes such as autophagy and apoptosis, based on the fact that physical exercise as a non-pharmacological strategy seems to rescue the imbalance between autophagy and apoptosis during aging. Therefore, the effects of different types or modalities of physical exercise in humans and animals, and the benefits of each of them on aging, will be discussed as a possible preventive strategy against neuronal death.

## Aging

The aging process is characterized by a series of molecular and cellular changes over the years that could culminate in the deterioration of physiological parameters important to keeping an organism alive and healthy. This widespread loss of body function, or loss of fitness, is extremely variable and can result in increased individual vulnerability, the onset of various illnesses, and death (1, 2). At the cellular level, aging is characterized by aggregation and accumulation of misfolded proteins and disabled organelles in a progressive way that may lead to cell homeostasis interruptions. Therefore, progressive degeneration may occur, increasing the risk of cell death [reviewed by (1, 3)].

In the Central Nervous System (CNS) normal aging is accompanied by alterations in brain structure such as white matter atrophy (4, 5), and functional and cognitive decline. It is still unclear what relationship the cognitive and functional dysfunctions have with the decrease in neurogenesis observed during aging (6). Age-related cognitive decline can reduce the quality of an individual's life and is related to an increased risk of neurodegenerative diseases (7, 8).

Neurogenesis consists in the generation of new neurons from neural stem cells and progenitor cells that reside in germinal niches in the subgranular zone (SGZ) of the hippocampal dentate gyrus and in the subventricular zone (SVZ) of the lateral ventricle (9). In relation to humans, neurogenesis is still a highly controversial topic, because of the inherent difficulty in marking neurogenic niches and newborn cells *in vivo* (10). Notwithstanding, a recent study raised the debate about the existence of neurogenesis in the human brain and its meaning. Analyzing 37 postmortem and 22 intraoperative tissue samples from human hippocampus, Sorrells et al. concluded that, different to other species, dentate gyrus (DG) proliferating progenitor cells and newborn neurons in humans decline during childhood without being detected in adult brain samples, suggesting a decline in neurogenesis during life (11). Besides, some studies suggest that neurogenesis happens daily in human DG (12–14), whereas others find a decrease in neurogenesis with just a few neurons being generated in adults (15, 16). Indeed, in 2019 new studies supported the evidence of hippocampal neurogenesis in adult humans, with great individual variability (17) up to the ninth decade of life in healthy subjects (18) and also in patients with mild cognitive decline and Alzheimer's Disease (17).

In animal models, physical exercise has been related to increased hippocampal neurogenesis (19, 20) which is reduced in old rodents (21), whereas in humans it is still speculative (22). However, in humans of all ages, physical exercise is related to improved memory function (23–25), as well as reduction in brain atrophy observed during aging in humans (26) and rodents (27). In the face of such an interesting approach against age-related cognitive decline, the focus of this review is to present and discuss some cellular mechanisms by which different kinds of physical exercise can unleash possible benefits in the CNS in human and animal models.

## Physical Exercise and Aging

Physical exercise was defined by Caspersen as a planned, structured and repetitive physical activity done with the objective of improving or promoting physical fitness (28). Exercise has been an important force of evolution for the human species in order to hunt for food sources, adapt to the environment and, in consequence, to alter physiology and development of the brain, displaying co-evolution of neuroplasticity signaling pathways (29–32).

The benefits of physical exercise can be observed throughout different stages of life. During pregnancy, supervised moderate exercise attenuates prenatal depression (33). It is also associated with a shorter first stage of labor (34), and newborns whose mothers exercised during pregnancy presented a better auditory memory response related to sound differentiation (35). Thus, an active lifestyle during pregnancy should be encouraged and promoted by public health policies (36). Among adolescents, physical activity may have beneficial effects on attention capacity and cognitive functions (37, 38), and is likely to be effective in reducing depression symptoms amongst both adolescents and young adults (39–42). Meanwhile, different modes of exercise are investigated for the older population, including stretching exercise, such as Pilates (43, 44) and Tai Chi Chuan (45, 46), resistance exercise (26, 47), multimodal exercise (48–50) and aerobic exercise (51). These modes of exercising seem to be similarly effective regarding cognitive improvement (23), but such improvement may not be seen in a short period of time (52).

Some studies and public organs recommend 150 min of moderate physical exercise per week to be sufficient for beneficial outcomes (53, 54). However, few elderly people accomplish the recommendation, especially of moderate to vigorous physical exercise; some believe physical exercise may be potentially harmful or even unnecessary [reviewed by (55)]. A review of nine cohort studies indicated that lower doses of the recommended physical exercise may reduce mortality risk by 22% (56), indicating that, even though it is believed that it is necessary to reach the suggested amount of activity, there is also the need to investigate whether light intensity exercise could ameliorate health or function and motivate the practice of more intense exercise (57).

Among the various interventions that affect aging, physical exercise seems to be the main ally in the prevention of aging-related diseases (58).

Studies regarding the effects of physical activity on elderly people also extend to several types of aging-related disorders, comprising dementia (59), late-life depression (60, 61), frailty syndrome (62, 63), Parkinson's Disease (64) and Alzheimer's Disease (65), through evaluation of epigenetic changes [see (66, 67) for review]. However, studies about the effects of physical exercise on elderly people's epigenetics are still emerging. Lavratti et al. conducted one of the first human studies to demonstrate the relationship between physical exercise and levels of global histone acetylation in schizophrenic patients (68). A meta-analysis on elderly people supports the protective effects of physical activity, a healthy diet and higher educational levels (69); however, in 2018, Gale et al. investigated the effect of physical activity on the epigenetic clock [reviewed in (69)] and found no correlation between them, indicating that exercise alone might not be enough to exert a protective effect in this specific regard.

It has already been demonstrated in the literature that physical exercise can promote neuroprotection. For example, treadmill physical exercise carried out in a mouse model of Alzheimer's disease (69) and voluntary running physical exercise in elderly mice (70) demonstrated, among other effects, that running physical exercise decreased glia activation and amyloid-beta (Aβ) peptide levels, suggesting possible mechanisms for exercise-induced neuroprotection.

The literature brings some ways in which physical exercise promotes its neuroprotective effects. In mice, it has been shown that neuroprotection induced by resistance physical exercise occurs in combination with multiple synergistic neuroprotective pathways: increased neurogenesis, decreased loss of dopaminergic neurons, increased antioxidant capacity, and improved autophagy (70). A study carried out in rats suggested that aerobic physical exercise reverted the synaptic loss in the cortex and hippocampus in old rats, which may be related to the up-regulation of Rho-GTPases (a G protein family, which plays a fundamental role in synaptic morpho-functional changes) (71). In mouse model of Parkinson's disease induced by MPTP, endurance physical exercise promoted neuroprotection possibly due to its contribution to the improvement of mitochondria biogenesis and reduction of apoptosis (72), decrease of pro-inflammatory cytokines and α-synuclein protein (73). In ischemic brain injury rat model, aerobic physical exercise can contribute to neuroprotection by blocking glia activation and preventing neuronal death (74).

Given the data presented here, it is likely that physical exercise is able to promote neuroprotective effects, which seem to depend on the type of physical exercise performed.

In addition to that, in a more general view about the effects of physical exercise in aging, there are many studies regarding cerebrovascular function, gut microbiota, hormone release, sleep quality, and neurotrophic factors production. These topics are quite large and complex and are beyond the scope of this review, however we will briefly mention the main data to contextualize the reader.

Cerebral blood flow (CBF) is the main marker of cerebrovascular function and its decrease, mainly due to detriment of energy depletion or brain ischemia [reviewed by (75)], seems to be related to the generation of cognitive impairment and dementia (72). Disruption of neuronal environmental homeostasis through impaired CBF can be highly related to the decline of the cerebrovascular system during aging (73, 74). In patients with Alzheimer's disease, in addition to decreased brain volume, there is also a decrease in CBF, and it is a potential marker of the severity of the disease (76).

In humans, physical exercise can prevent cognitive impairment by enhancing cerebral vasomotor reactivity, increasing CBF, and consequently increasing cerebrovascular function in older adults (77, 78). Such an increase seems to be dependent on exercise intensity (79). In sedentary older men, aerobic physical exercise was able to increase CBF in the region responsible for regulating cognitive functions, part of this mediated by improvements in glucose metabolism (78). Moreover, another study involving sedentary older men verified the increase in brain function mediated by the regional CBF, increased cognition assessed by memory and executive functions, and the increase in cardiovascular fitness measured by VO2 max, after a protocol of 12-week exercise (80). Otherwise, a study with master athletes ranged in age between 50 and 80 demonstrated that the exercise cessation for a short period of time reduced CBF levels in hippocampus and gray matter regions (81).

In animals, physical exercise through treadmills or running wheels was able to improve endothelium-dependent vasorelaxation as well as increase CBF, reducing functional deficits and protecting the brain from cerebral ischemia and reperfusion (82).

Regarding the gut-brain axis, it is known that gut microbiota plays important roles on metabolic and immunological activities in humans (83). Infrequent bowel movements can decrease gut microbiota, which increases the risk of individuals developing colorectal cancer (84, 85). A combination of moderate physical exercise [≥ 7000 steps/day or 15 min/day at >3 METS (metabolic equivalents)] and lactobacillus ingestion has been shown to decrease infrequent bowel movements in elderly people aged 65–92 (86). In elderly humans, it was shown that 5-week endurance physical exercise was not able to significantly change gut microbiota diversity and composition (87). In the animal model, it has been shown that 11-month-old mice submitted to treadmill physical exercise for 7 months had their gut microbiota diversity augmented, suggesting that physical exercise is able to increase microbiota diversity during the aging process (88). However, Fielding et al. found that mice presented no changes either in their entire lean body mass or in treadmill endurance capacity when treated with human feces coming from elderly people who exercised. Therefore, data regarding physical exercise influence on gut microbiota along aging seems contradictory both in animals and humans, likely depending on duration, intensity and type of physical exercise (89).

It is well-established that growth hormone (GH) secretion decreases during aging process (90–93), which seems to be associated with changes in the organism such as loss of lean mass, gain of fat tissue, diminution in muscle strength, decline in cognitive function, among others [reviewed in (94)]. Physical exercise in the elderly has been described to influence GH level/activity; studies in humans, independently of gender, have demonstrated that regular physical exercise can increase GH levels in plasma (95, 96) or serum (97–103).

However, other studies have not shown an increase of GH levels in elderly marathon runners and sedentary controls (104), in middle-age men (40–50 years old) (105), in old men (47), in old women (106) and in old men and women. The comparison was conducted after subjects had been submitted to heavy resistance training (107), low volume resistance exercise (108), and low intensity physical exercise (109).

Furthermore, experiments done in 21-month-old rats showed that mild physical exercise in treadmill (8 m/min, 1 h/day) in combination with GH administration for 73 days, increased both muscle mass and strength compared with GH by itself (110). However, Marzetti et al. showed that short-term treadmill training attenuates age-related skeletal muscle apoptosis and the same effect was not observed with short-term administration of GH in older rats (111). Studies performed in old rats showed that exercise and GH reduced age-related decay in myocardial relaxation, avoiding diastolic dysfunction (112) and increasing bone strength (113, 114). It is known that GH can augment muscle mass in humans (92). In humans, administration of GH and testosterone together in elderly males produced a gain in lean mass and increased muscle strength, and consequently aerobic endurance (115). Another study about GH supplementation in elderly men did not observe increased muscle strength, and consequently no changes in resistance exercise (116).

Sleep disturbances are common features in older adults, such as sleepiness at daytime, fractionated sleep at night (117–119), among others. It has been shown in the elderly, both men and women, that low to moderate physical activity improved sleep quality (120–133). In animals, sleep derangement related to the aging process also happens (134, 135). It seems that regular moderate physical exercise ameliorates sleep architecture in old rats (136).

A great number of signaling pathways seem to be involved in physical exercise benefits, and one of them is brain-derived neurotrophic factor (BDNF) which is positively induced by physical exercise (137). BDNF is a protein that participates in neuronal proliferation and differentiation, synaptogenesis, synaptic function, plasticity, and neuroendocrine actions [see Review (138)]. In 1995, Neeper et al. measured BDNF mRNA in different brain regions of adult rats with different levels of physical activity, and found a positive correlation between the distance run per night and the BDNF produced in the hippocampus and caudal neocortex of these animals (139). Moreover, BDNF acts as a regulator of the ubiquitin-proteasome system (UPS) as it increases ubiquitin conjugation in synaptic proteins during synaptic remodeling. In addition, the use of a proteasome pharmacological inhibitor prevented BDNF-mediated action and had the same profile as the BDNF signaling block (140).

In summary, the benefits of physical exercise can be observed throughout different stages of life. Especially during aging, neuroprotection is promoted according to type and intensity of physical exercise. In general, it improves cerebrovascular health and gut microbiota diversity, which seems to be related to healthy aging. Furthermore, physical exercise improves quality of sleep and increases BDNF production, and can decrease neuronal death and improve cognitive performance, due to better functioning of the proteostasis system, among many other effects. Besides, data regarding physical exercise influence on gut microbiota, GH, CBF during aging seems contradictory both in animals and humans, likely depending on duration, intensity and type of physical exercise.

## Proteostasis and Aging

**Figure 1:**
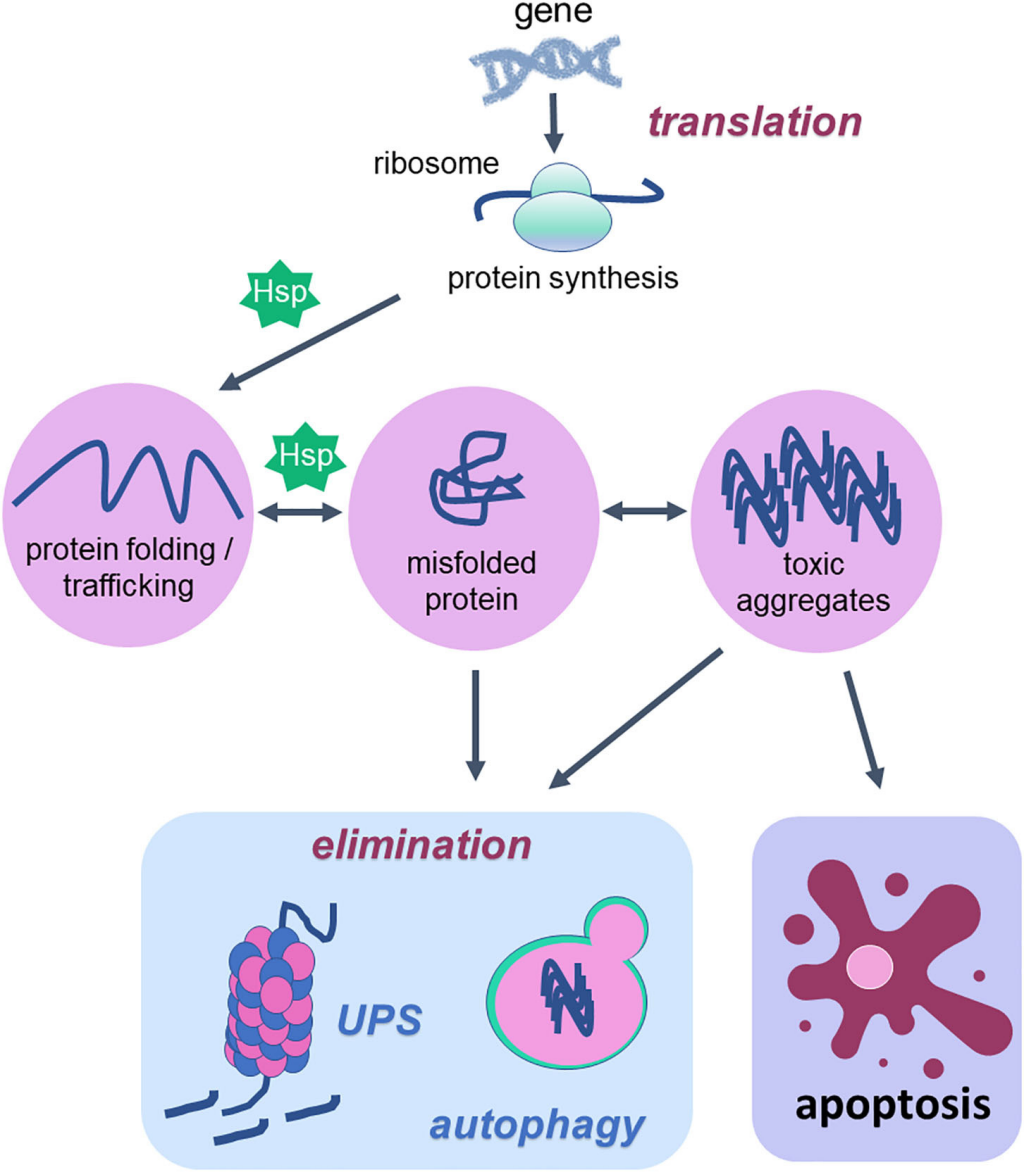
Cellular proteostasis and apoptotic cell death. Proteostasis network is the protein pool quality control system that integrates the pathways related to protein synthesis since translation, protein folding, protein unfolding, secretion, trafficking and degradation or elimination. To succeed in protein folding, unfolding, refolding and trafficking, chaperone proteins (heat shock proteins—Hsp) are of fundamental importance. In the elimination phase that usually happens when misfolded proteins turn into toxic aggregates, degradation of damaged proteins occurs through proteolytic systems: autophagy and ubiquitin-proteasome. The activity of both systems avoids cell death. However, when the proteostatic network cannot avoid protein aggregates accumulation, the cell undergoes apoptosis. Thus, degradation system and apoptosis are both important mechanisms for organism homeostasis due to the elimination of damaged proteins and damaged cells, respectively.

The mammalian protein pool is subject to a constant quality control system that integrates the pathways related to protein synthesis, folding, unfolding, secretion, trafficking and degradation (Figure 1). This quality control system is known as proteostasis and its failures rely on increased levels of protein aggregates, which contribute to the development of proteinopathies and thus neurodegenerative diseases including Alzheimer's disease, Parkinson's disease, and amyotrophic lateral sclerosis (141, 142). The proteostasis decline is one of the hallmarks of aging (1, 143) and this decline can be explained by increased generation of oxidative damage within the cells (144).

**Figure 2:**
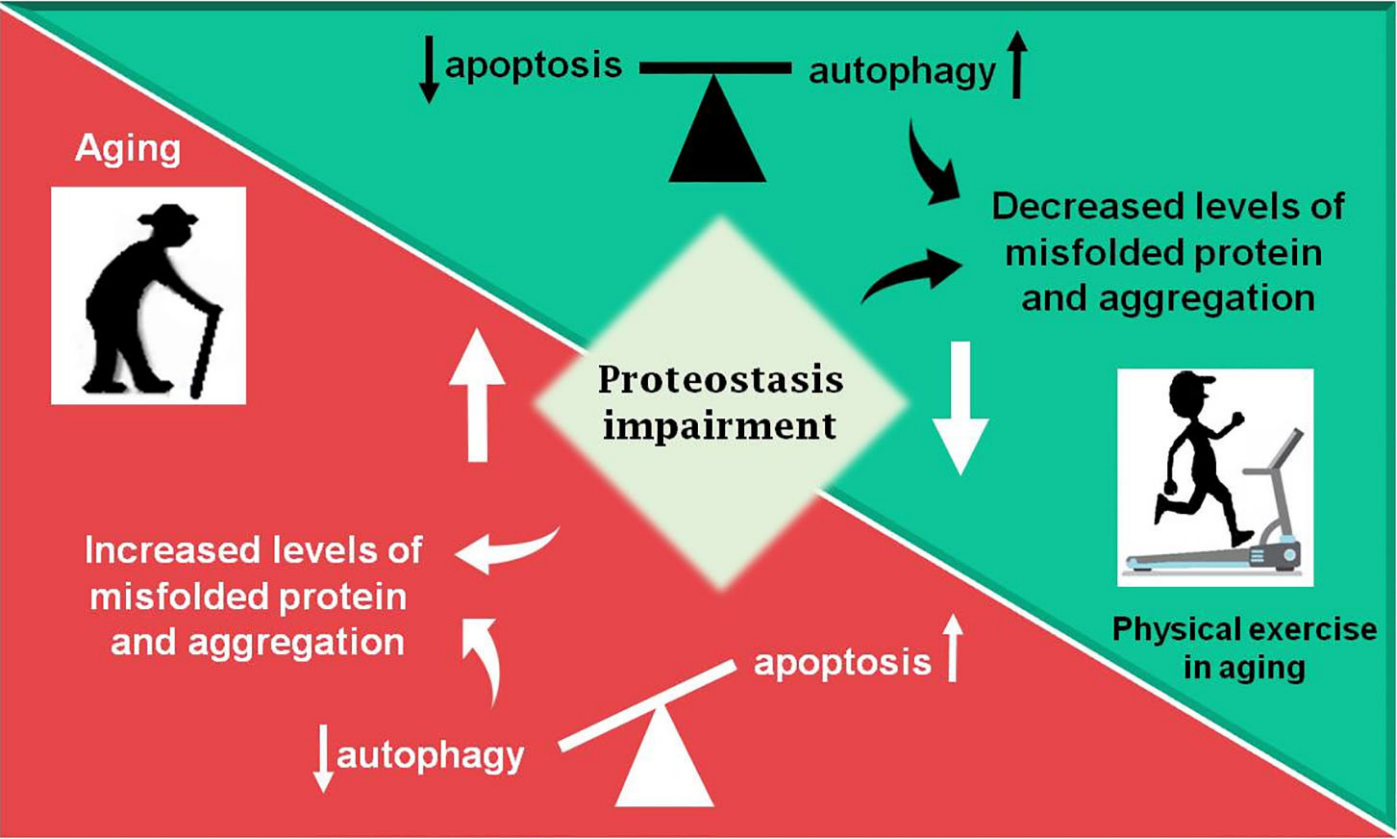
Summary of the review. During aging (red box) an increase in proteostasis impairment is observed in the Central Nervous System, which may lead to increased levels of misfolded proteins, in part due to decreased autophagic process. The balance between apoptosis and autophagy is lost and an increase in programmed cell death is observed. Otherwise, physical exercise in aging (green box) can partially revert the disbalance observed in aging, decreasing proteostasis impairment and improving autophagic process, as well as decreasing levels of misfolded proteins and toxic protein aggregates, which lead to less apoptosis activation.

According to a vast literature in the field, the proteostasis network is mediated by the degradation of damaged proteins by proteolytic systems (autophagy and ubiquitin-proteasome) and correction or sequestration by chaperones (141, 145, 146). Although proteostasis involves all of these processes, this review will focus on the balance between two vital cellular processes such as autophagy and programmed cell death, apoptosis, based on the fact that there are several studies done with physical exercise as a non-pharmacological strategy that rescues the lost balance between them during aging (Figure 2).

The ubiquitin-proteasome system (UPS) is extremely important for the maintenance of protein homeostasis in the cytosolic and nuclear compartments. Ubiquitination occurs through catalytic enzymes, E ligases, which activate ubiquitin and covalently bind this polypeptide to the substrate, a tag for proteasome degradation. The proteasome, or 26S, is a multicatalytic complex with a proteolytic core, 20S, flanked by regulatory units that recognize the ubiquitinated substrates, misfolded and damaged proteins or healthy proteins, and lead them to degradation (147, 148). During aging, this system may be compromised due to defective proteasome activity, proteasome damage, proteasome assembly changes and ubiquitination defects (141). Studies with *Drosophila melanogaster* demonstrated a change from the 26S “activated” proteasome (1–32 days old) to the weakly active 20S form (43–47 days old) during aging, together with decline in ATP levels, highly necessary for the 26S proteasome activity (149). Therefore, UPS is one of the proteolytic systems that degrades damaged proteins regulating the proteostasis network (150, 151).

## Autophagy in Aged Brain

During the aging process, organelles and proteins are prone to damage affecting their normal functionality, besides that these dysfunctional proteins, and organelles accumulate in the body progressively, thereby increasing the rate of cell death (1, 3). Studies indicate that the loss of autophagic activity in cell aging contributes to a progressive reduction in cell function and may precipitate cell death by restricting the ability of cells to support a healthy population of proteome and organelles (152, 153). Aging is associated with reduced autophagy potential and it has been shown in the literature that autophagic inhibition may result in premature aging (153).

Autophagy (divided into macroautophagy, microautophagy, and chaperone-mediated autophagy) is an important process of cell renewal in maintaining homeostasis and perfect cellular functionality, characterized by the elimination of non-functional proteins, damaged/defective organelles, and intracellular pathogens. Autophagy is an important cell survival mechanism with an important role in cell maintenance and homeostasis and with a positive influence on useful life and longevity (153–159).

**Figure 3:**
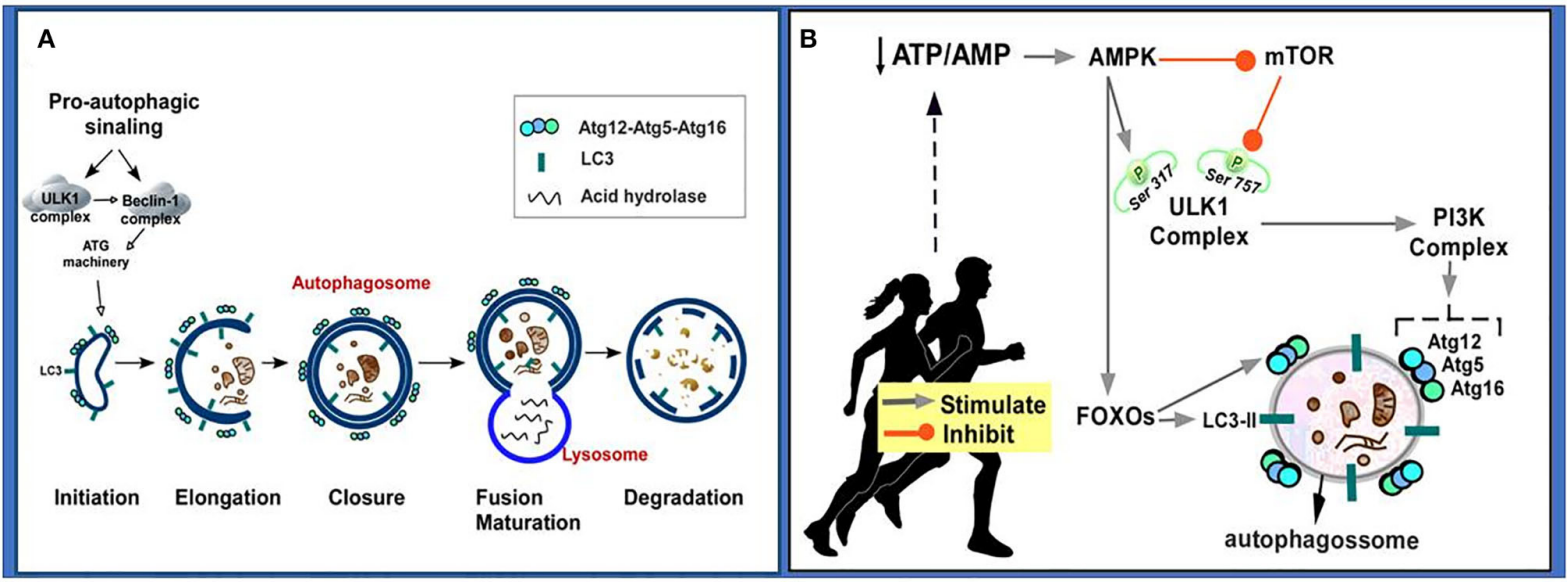
Autophageal mechanism and stages. **(A)** Pro-autophagic signaling induces ULK1 activation and Beclin-1 complex, activating the ATG machinery, thereby initiating the autophagy stages consisting of initiation, elongation, closure and autophagosome formation, autophagosome fusion with lysosome and degradation. **(B)** Physical exercise causes a decrease in the ATP/AMP ratio by activating AMPK. AMPK inhibits the mTOR pathway, so ULK Ser^757^ phosphorylation will be decreased and it may interact with AMPK and be activated by phosphorylation on Ser^317^. AMPK phosphorylated ULK1 becomes active and may initiate autophagy. PI3K activation occurs culminating in increased expression of autophagy-related proteins (Atg12, Atg5, Atg16). AMPK also promotes signaling of autophagy-related transcription factors (FOXOs), thereby increasing the expression of autophagy-related proteins (Atg12, Atg5, Atg16, LC3-II). Atg, autophagy-related protein; ATP/AMP, adenosine triphosphate/adenosine monophosphate; AMPK, AMP-activated protein kinase; Beclin-1, autophagy-related protein; FOXO, Forkhead box O; LC3II, microtubule-associated protein 1 light chain 3–form II; mTOR, mammalian target of rapamycin; PI3K, Phosphatidylinositol 3-kinase; UKL1, Unc-51-like kinase 1.

The macroautophagy, here referred to as autophagy, has been the most studied. In summary, when damaged proteins and/or organelles are free in the cytoplasm, a nascent membrane originated from Golgi Complex, endoplasmic reticulum (ER), mitochondria, plasma membrane or endosomes is formed to engulf and sequester these damaged elements. This primordial membrane is called phagophore, which will, in a second step, fuse at its edges forming a double-membrane vesicle called autophagosome. The autophagosomes will undergo a maturation step in which they fuse with acidified lysosomal or endosomal vesicles to finally degrade a damaged element and recycle it (Figure 3A) (160, 161).

Among the main proteins that control the autophagic process, we can mention: autophagy-related (Atg) protein, which is associated with cytosolic component sequestration and autophagosome formation and is crucial for normal autophagic function (162, 163), for example Atg5, Atg12, Atg16; LC3, which participates in the phagophore and autophagosome expansions (164, 165); and Beclin-1, protein which participates in the initiation of the autophagic process by interacting directly with the phosphatidylinositol 3-kinase (PI3K) complex (166, 167). Autophagy is a very tightly controlled process that can adapt cellular metabolism to a stressful situation, such as starvation and growing factors deprivation, and can also be involved in turnover of organelles and long-lived proteins. Thus, autophagy and apoptosis are both important mechanisms for organism homeostasis due to the elimination of damaged or superfluous cellular components and damaged cells, respectively (160).

Autophagy dysfunctions may contribute to neurotoxicity associated with neurodegeneration and aging (168). Decreased age-related autophagy disrupts neuronal homeostasis and may thus promote the process of neurodegenerative disorders (169–171). However, data in the literature indicates that exercise can activate autophagy, thus preventing age-related diseases as well as retarding neurodegenerative processes [see reviews for more information (161, 172)].

Atgs knockout experiments have shown defects associated with aging, such as high accumulation of non-functional organelles (173–175), endoplasmic stress (173) and mitochondrial disorder (174–176). However, it remains unclear whether these Atgs reductions are, in fact, the main reason for age-related autophagic malfunction. Literature data suggests that basal autophagy decay may be mediated by excessive activity of rapamycin complex 1 (TORC1), a protein kinase that negatively regulates autophagy. The literature has shown that inhibition of TORC1 may increase longevity (177–179).

## Influence of Physical Exercise in the Autophagy Process in Aged Brain

It has been postulated that regular physical exercise can promote a beneficial effect on the health of individuals and is considered an important autophagic inducer (180–183). It was observed that treadmill exercise (8 weeks) in mice modulated the levels of autophagy-associated proteins, including Beclin1, and improved autophagy (184). Based on literature data, it is suggested that physical exercise can induce autophagy through the following mechanism: exercise induces decreased adenosine triphosphate/adenosine monophosphate (ATP/AMP) in the cell, and this induces AMP-activated protein kinase (AMPK) activation; AMPK activation promotes inhibition of mammalian target of rapamycin (mTOR), leading to Unc-51 kinase 1 (ULK1) disinhibition, which is also phosphorylated and activated by AMPK; the ULK1 complex induces activation of the Phosphatidylinositol 3-kinase (PI3K) complex culminating in increased expression of autophagy-related proteins (Atgs); AMPK also promotes activation of autophagy-related transcription factors such as forkhead box O (FOXO), thereby increasing the expression of LC3-II and Atgs [for more detailed information on these signaling pathways, see (172, 185, 186) (Figure 3B)].

Based on data published in the literature, it is possible to suggest that the induction of autophagy by stimulating physical exercise is regulated according to exercise type, duration and/or intensity-dependent manner (182).

Kou et al. noted that swimming can delay the aging process, rescuing the impaired functional state of autophagy and abnormal mitochondrial dynamics. In addition, Luo et al. observed that 10-week swimming exercise in rats promoted adjustments in lysosomal degradation, activation of autophagy and mitochondrial quality control in the hippocampus, preventing age-associated cognitive decline. These findings indicate that the conservation of cognitive function in older rats by exercise is associated with mitochondrial improvement in the hippocampus, and lysosomal degradation is required in this process, suggesting that exercise and lysosomal degradation may be effective in decreasing age-related cognitive decline (187).

Huang et al. show that 8-week running exercise in mice can activate the autophagy pathway and improve lysosomal biogenesis, suggesting improvement in brain function of mice; besides that, they also observed that prolonged physical exercise promoted nuclear translocation of transcription factor EB (TFEB–main regulator of autophagic and lysosomal biogenesis) in the cortex, positively regulating the transcription of genes associated with autophagy and lysosome (lysosomal degradation is a fundamental step to completing the autophagy process) (188). It has also been observed that moderate exercise contributes to the prevention of early neurodegeneration in the substantia nigra region in aged rats by improving autophagy and mitophagy (189).

Given the data mentioned here, we can conclude that during aging there are dysfunctions in autophagy leading to CNS damage. Physical exercise could attenuate or prevent such autophagic dysfunctions. However, further studies are still necessary to indicate which modality, duration and intensity of physical exercise induce the greatest positive effects on CNS autophagy.

## Apoptosis in Aged Brain

**Figure 4:**
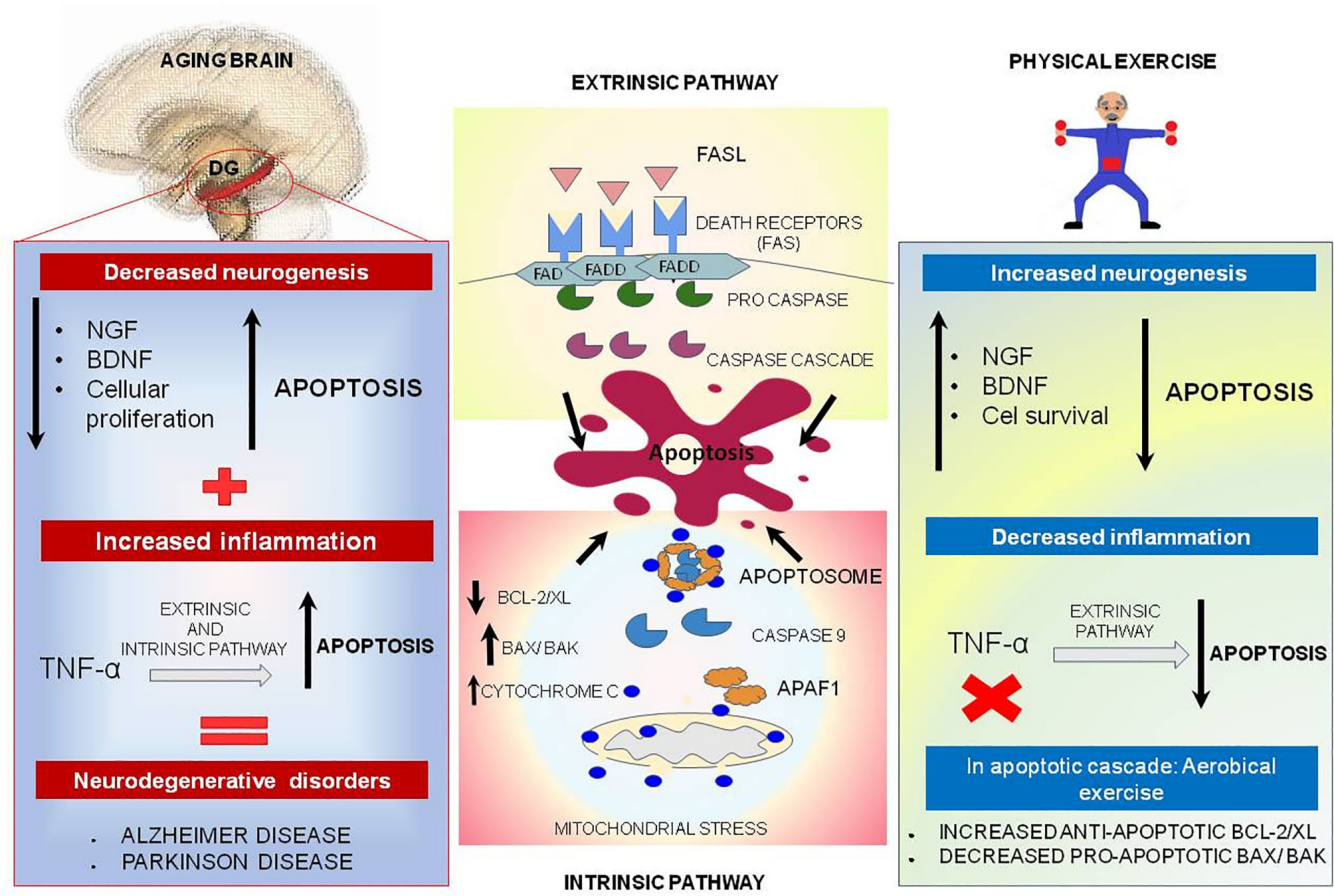
Physical exercise actions against aging brain and the apoptotic process. In the central boxes: Apoptosis can be divided into extrinsic and intrinsic signaling pathways. The extrinsic pathway illustrated in the upper central box is induced by death receptors such as FASL, and their ligands, FAS. On the other hand, the intrinsic pathway illustrated in the central inferior box is induced by signs that cause changes in the mitochondrial membrane, called mitochondrial outer membrane permeabilization (MOMP). This process generates macropores on the mitochondrial surface and a release of Cytochrome C, which once released by mitochondria binds to and induces APAF-1 and pro-caspase 9, forming the Apoptosome complex, which activates caspase 9 causing cell death. This pathway is mediated by anti-apoptotic proteins such as BCL-2 and BCL-XL, and pro-apoptotic proteins such as BAX and BAK. In the left box, brain aging is illustrated, and a decrease of neurotrophic factors such as nerve growth factor (NGF) is observed, and brain-derived neurotrophic factor (BDNF), especially in dentate gyrus located on hippocampus, may be related to decreased cell proliferation and increased apoptosis. In addition, TNF-alpha, a pro-inflammatory cytokine, can increase the apoptosis process by intrinsic and extrinsic pathways and lead to the emergence of neurodegenerative diseases. Physical exercise effects, illustrated in the right box, act in reverse, increasing the amount of NGF and BDNF, increasing cell survival and culminating in decreased apoptosis. In addition, physical exercise inhibits the production of pro-inflammatory cytokine TNF-alpha which, in low quantities, decreases the extrinsic apoptosis process.

Apoptosis is a process of programmed cell death modulated by the B cell leukemia/lymphoma 2 (Bcl-2)/Bcl2 associated X protein (Bax) family and upregulated during the aging process (190, 191), which is important for tissue homeostasis (192). Apoptosis basically occurs in two different pathways: extrinsic and intrinsic. The extrinsic pathway is induced by death receptors and their ligands (Fas/ FasL complex) or via pro-inflammatory marker (tumor necrosis factor (TNF)α), and the intrinsic pathway is regulated by mitochondrial stress which activates caspase 9 and cleaves caspase 3 (Figure 4) (193, 194).

The Bcl-2 family is related to apoptotic intrinsic pathway and has a range of 20 different proteins. Each protein of this family has homology domains: BH1 to BH4. Pro-apoptotic proteins are related to BH3 domains called BCL2 antagonist killer 1 (BAK) and BAX. The other domains are related to anti-apoptotic proteins, known as BCL2, BCLXL, BCL2L2, myeloid cell leukemia 1 (MCL1) and BCL2A1 (194–196). In the intrinsic pathway an increased expression of anti-apoptotic Bcl-2 proteins modulates the expression of cell cycle inhibitors and induces cellular senescence, while the expression of pro-apoptotic factors such as Bax and Bak proteins results in macropores formation in the mitochondrial surface, called mitochondrial outer membrane permeabilization (MOMP). This pore allows the exit of cytochrome c from mitochondria, which culminates in activation of caspase cascade causing cell death. Bcl-2 acts as an anti-apoptotic factor that can inhibit the activation of Bax or Bak and inhibits autophagy by beclin-1, which modulates cellular senescence (194–197). Caspase-3 is one of the key proteins of apoptosis and could be responsible for the proteolytic cleavage of many proteins such as poly (ADP-ribose) polymerase (PARP) responsible for DNA repair observed by Cechella et al. in aged rat brains (27).

In the aged brain, there is a reduction in the availability of neurotrophic factors such as nerve growth factor (NGF) and brain-derived neurotrophic factor (BDNF), especially in the hippocampus; therefore, these changes may be linked with the large reduction in cell proliferation and increase in apoptosis in the dentate gyrus (8, 198). Besides, TNF-α, a pro-inflammatory protein, could increase the process of apoptosis via intrinsic and extrinsic pathways, which is more significantly visible in aged brain and could be implicated in neurodegenerative diseases, such as Alzheimer's disease (AD) and Parkinson's disease (PD) (Figure 4) (199–201).

## Influence of Physical Exercise in the Apoptosis Process in Aged Brain

Sedentary lifestyle could be a risk factor for cognitive dysfunction and neurodegenerative process, and regular exercise has anti-aging effects, more specifically in the CNS, whose benefits include an increase in hippocampus neurogenesis, which improves learning and memory in aged rodents. Exercise can upregulate BDNF in hippocampal and cortical neurons promoting synaptic remodeling and improving cell survival (202, 203). Physical exercise inhibits the production of the pro-inflammatory cytokine TNF-α, which in low concentration decreases the process of apoptosis via the extrinsic pathway (200).

Aerobic Physical Exercise (APE) is the most common type of training for rodents, such as running and swimming, and they must be conducted repeatedly with an established frequency; however, few studies have compared the effects of many different modes of exercise on cognition (7). APE is able to positively affect the dynamic adaptations of the neuronal terminal zones in aging. Fattoretti et al. observed effects of APE in the hippocampus of old mice (27 months) submitted to a 4-week aerobic training in a treadmill apparatus, five times per week, and found that hippocampal regions are not uniformly influenced by physical training: they show an increase in the number of synapses and synaptic area per μm^3^ of tissue in CA1 caused by physical exercise protocol in aged animals, while in DG they could only see an increased number in the synaptic area (204).

Moderate intensity APE protocol for 28 days in 18-month-old mice induced an upregulation of hippocalcin, α-spectrin and ovarian tumor domain-containing ubiquitin aldehyde-binding protein 1 (OTUB1) in the hippocampus (6). Hippocalcin, which is a calcium ligand, has been shown to protect neurons against apoptosis by regulating inhibitory proteins (205). Xie et al. using an intracerebral hemorrhage method in rats, verified that OUTB1 colocalized with active caspase 3 and attenuated neuronal apoptosis (206). Besides, hippocalcin and spectrin-α in the hippocampus of old mice could be related to an increase in neurogenesis (7).

Fang et al. tested a 12-week protocol of treadmill aerobic exercise in aged rats, and observed by TUNEL staining and immunohistochemistry that the number of TUNEL positive cells (dead cells) had diminished in cortex and hippocampus after the exercise protocol. Moreover, anti-apoptotic protein Bcl-2 levels were augmented. On the other hand, pro-apoptotic proteins, Bax and cleaved caspase-3 levels were decreased in hippocampus and cortex of these rodents submitted to the APE protocol. Another important evidence for the benefits of APE is the decrease of the cytoskeletal protein tau hyperphosphorylation in the brain of old rodents (197, 207–209).

APE is not restricted only to the treadmill apparatus. Cechella et al. submitted rats to a forced swimming protocol for 4 weeks and one experimental group of rodents were supplemented with diphenyl diselenide ((PhSe)_2_), a potential antioxidant compound. Both groups, exercise and exercise plus (PhSe)_2_, could decrease the level of pro-apoptotic proteins in 27-month-old rats. They observed an increase in BDNF levels that has led to a downregulation of pJNK/JNK ratio which induced caspase 3 cleavage. The cleaved caspase 3/caspase 3 ratio was also decreased, while Bcl2 expression was increased in animals submitted to both protocols (27). Conversely, Liu et al. affirmed that, after 10 weeks of treadmill exercise, apoptotic striatum cells in old-aged rats increased by 68.24% in comparison to sedentary animals, showing that intense physical activity might not be beneficial for the organism (210).

Do et al. tested voluntary running in triple transgenic AD mice for 4 and 8 weeks. At the end of this period, they showed a reduction of apoptotic cells in the hypothalamus (211). In addition, 4-week voluntary exercise training reduced pro-inflammatory cytokines TNF-α and IL-6 to the levels compared to the control, which supports previous studies in the literature (212, 213).

There is a great amount of research related to aerobic exercise and apoptosis, but little is known about the effects of resistance training (RT) on the brain. Aerobic exercise may induce events such as cell proliferation, survival and death through distinct mechanisms from those of resistance exercise (214, 215). The RT consisted of bodybuilding exercises like adductor, abductor, leg presses, triceps and biceps exercises, or in a simpler way for rodents, it could be climbing a ladder repeatedly, for example (200, 216, 217). de Smolarek et al. (217) showed that RT could be very suitable for the elderly population because they were able to see an improvement in cognition in elderly women (between 65 and 69 years old) submitted to resistance training for 12 weeks. Another study that used RT protocol in elderly women (between 65 and 75 years old) for 52 weeks showed, after this period, an increase in these women's cognition, mainly in learning, measured by Mini-Mental State Examination and another verbal quiz (26).

There are few studies in the literature showing that RT could be used for hospitalized elderly people who spend most of the time lying on a bed relying exclusively on physiotherapy exercises (218–221). Martínez-Velilla et al. used an RT protocol that includes physical exercises such as line walking, stepping practice, proprioceptive exercises, among others (219). The results showed that RT protocol was efficient in preventing functional decline caused by hospitalization (218). Other clinical trials with RT protocol applied in 65-year-old healthy men and women, consisting of a 3-month body-mass-based exercise program, revealed that only working memory was improved (222).

Frailty is a syndrome characterized by dysregulation of several physiological systems (223, 224). Few studies with physical exercise (not only RT exercise, but also APE) were conducted to evaluate its efficiency in ameliorating frailty phenotype (218, 225). Yoon et al. tested an RT high speed program in elderly people (74 years old) with frailty syndrome for 16 weeks and they observed that RT improved cognitive and physical functions (218). Based on clinical trials data, we can conclude there is a good outcome in using RT protocol in the elderly to improve their cognition (218, 219, 222).

In another perspective, regarding animal studies, Henrique et al. (200) compared two protocols, APE and RT. APE protocol used treadmill running, and RT protocol consisted of a series of eight climbs with a progressively heavier load, both for 7 weeks in 21-month-old rats. They observed that the RT group had a reduction in hippocampal levels of macrophage inflammatory peptide (MIP)-2 protein, a pro-inflammatory mediator which seems to be related to inducing apoptosis (226), so the effects of RT on MIP-2 levels deserve to be more explored.

Vilela et al. (215) also compared APE and RT in 24-month-old rats and observed, in both protocols, an increase of the hippocampal neurotrophin receptor P75 (P75^NTR^), a transmembrane receptor involved in many cellular functions including apoptosis, cell survival, neurite outgrowth, migration, and cell cycle arrest (227, 228). In this case, the researchers believed that P75^NTR^ was involved in neuroprotection through activation of the apoptosis pathway to induce death process on damaged neurons and to provide a conducive environment for insertion of new cells (215, 229).

In summary, both APE and RT could improve spatial memory or activate different mechanisms that lead to cell survival and induce a decrease of apoptotic cells. RT can be a strategy to protect the brain and maintain healthy cognition during the aging process, while APE could alter intracellular pathways, even though the related mechanisms that explain these effects still remain unknown.

## Conclusion and Perspectives

Based on what we cited in this review, we can conclude that during the aging process dysfunctions can occur in several cellular events, such as autophagy and apoptosis, which can culminate in CNS damage. Physical exercise could attenuate or prevent such autophagic or apoptotic dysfunctions. At the same there is controversial data from the literature both in human and animal studies. Therefore, further studies are still necessary to clarify the effects of physical exercise during the aging process and also to demystify underlying mechanisms of physical exercise effects and indicate which modality, duration and intensity of exercise is able to induce the greatest positive effects in the CNS, thus preventing neuronal death.

## Author Contributions

EK conceived the review idea and edited the final version of the text and figures. JS, AO, AM, and DA contributed equally by writing the text as well as drawing the figures, and participated in the discussion of the review idea. PM contributed by drafting the review and drawing the figures. All authors contributed to the article and approved the submitted version.

## Conflict of Interest

The authors declare that the research was conducted in the absence of any commercial or financial relationships that could be construed as a potential conflict of interest.
